# *De novo* phosphorylation of H2AX by WSTF regulates transcription-coupled homologous recombination repair

**DOI:** 10.1093/nar/gkz309

**Published:** 2019-05-02

**Authors:** Jae-Hoon Ji, Sunwoo Min, Sunyoung Chae, Geun-Hyoung Ha, Yonghyeon Kim, Yeon-Ji Park, Chang-Woo Lee, Hyeseong Cho

**Affiliations:** 1 Genomic Instability Research Center, Ajou University School of Medicine, Suwon, South Korea; 2 Department of Biochemistry and Molecular Biology, Ajou University School of Medicine, Suwon, South Korea; 3 Institute of Medical Science, Ajou University School of Medicine, Suwon, South Korea; 4 Department of Molecular Cell Biology, Sungkyunkwan University School of Medicine, Suwon, South Korea

## Abstract

Histone H2AX undergoes a phosphorylation switch from pTyr142 (H2AX-pY142) to pSer139 (γH2AX) in the DNA damage response (DDR); however, the functional role of H2AX-pY142 remains elusive. Here, we report a new layer of regulation involving transcription-coupled H2AX-pY142 in the DDR. We found that constitutive H2AX-pY142 generated by Williams-Beuren syndrome transcription factor (WSTF) interacts with RNA polymerase II (RNAPII) and is associated with RNAPII-mediated active transcription in proliferating cells. Also, removal of pre-existing H2AX-pY142 by ATM-dependent EYA1/3 phosphatases disrupts this association and requires for transcriptional silencing at transcribed active damage sites. The following recovery of H2AX-pY142 via translocation of WSTF to DNA lesions facilitates transcription-coupled homologous recombination (TC-HR) in the G1 phase, whereby RAD51 loading, but not RPA32, utilizes RNAPII-dependent active RNA transcripts as donor templates. We propose that the WSTF-H2AX-RNAPII axis regulates transcription and TC-HR repair to maintain genome integrity.

## INTRODUCTION

DNA damage defense mechanisms protect the genome against various deleterious agents. In the DNA damage response (DDR) pathway, post-translational histone modifications play an essential role in regulating DNA damage signaling and repair (1,2). Phosphorylation of histone H2AX at Ser139 (γH2AX) is a well-known modification that regulates the DNA damage signaling pathway in an ATM- and ATR kinase-dependent manner (3–5). DNA double-strand breaks (DSBs) trigger the spreading of γH2AX domains flanking break sites, a process that protects against mutations and chromatin rearrangements (6). In mammals, phosphorylation of H2AX at Tyr142 (H2AX-pY142) is constitutively maintained by the tyrosine kinase activity of the chromatin remodeler Williams–Beuren syndrome transcription factor (WSTF) (7). Following DNA damage, the Tyr142 phosphorylation is removed by the ATM/ATR-dependent phosphatases eyes absent homologs 1 and 3 (EYA1/3) (8). In the DDR, dual phosphorylation of H2AX at Tyr142 and Ser139 results in partial apoptotic cell death. Consequently, dephosphorylation of H2AX-pY142 is important for proper functioning of the γH2AX-dependent DNA damage signaling pathway. Meanwhile, H2AX in cells is concentrated on the transcription start site and γH2AX enrichment upon irradiation also coincides with actively transcribed regions (9). However, the phosphorylation switch from H2AX-pY142 to γH2AX that links to transcriptional regulation is not established.

Transcriptional silencing in the DDR is tightly regulated by ATM kinase and histone modifications by Polycomb group proteins and the NuRD complex (10–14). Furthermore, the formation of γH2AX foci inhibits RNA polymerase II (RNAPII)-mediated transcription in active chromatin regions to maintain genome integrity (6,15). Recently, it was reported that active transcription also enhances transcription-coupled DSB repair, which occurs in a cell cycle-dependent manner (16). In the G2 phase, RNAPII-mediated histone H3 trimethylation at Lys36 (H3K36me3) at active genes recruits the transcriptional cofactor lens epithelium-derived growth factor p75 splicing variant via CtIP, allowing the initiation of resection and transcription-coupled homologous recombination (TC-HR) repair, using sister chromatids as a donor template. However, although the absence of sister chromatids indicates that classical non-homologous end-joining (c-NHEJ) is the major component of DNA repair in G1, the specific repair events that occur at active genes in this phase are still unclear. Recently, a role of active RNA transcripts in DNA damage signaling activation and efficient repair has emerged (17–19). Notably, Lan's group reported that DNA damage-induced active RNA transcripts trigger TC-HR repair through functional interaction with Cockayne syndrome protein B in the G0/G1 phase (19). Furthermore, RNAPII activity is required for formation of c-NHEJ repair factor 53BP1 foci and DNA repair via interaction with damage-induced RNAs and the MRN complex at DSB sites, although the cell cycle dependency of this process has not been investigated (18). Overall, coordination of transcription machineries and DNA repair factors promotes DNA damage surveillance and genomic integrity, but the exact mechanisms involved remain to be elucidated.

Here, we show that formation of H2AX-pY142 by WSTF is tightly associated with RNAPII and transcriptionally active histone marks at transcribed active sites in normal cells. We also demonstrate that removal of pre-existing H2AX-pY142 via ATM-dependent EYAs is required for transcriptional silencing at transcribed active damage sites. Finally, *de novo* phosphorylation of H2AX-Y142, mediated by translocation of WSTF to DNA breaks, is important for TC-HR repair via RAD51 recruitment and recognition of active RNA transcripts as templates in the cell cycle-dependent manner.

## MATERIALS AND METHODS

### Cell lines and chemicals

The human U2OS, U2OS 2-6-3, HEK 293T, HeLa, and HeLa H2AX knock-out cell lines were cultured in DMEM with 10% (v/v) FBS (Gibco) at 37°C. U2OS 2-6-5 cell was cultured in DMEM with 10% (v/v) FBS (tetracycline free; Gibco) at 37°C. The mouse embryo fibroblast NIH3T3 cell was maintained in DMEM/F-12 with 10% (v/v) FBS (Gibco) at 37°C. Plasmids and/or siRNAs were transfected with Lipofectamin2000 (Invitrogen) and/or RNAiMAX (Invitrogen), respectively. The RNA polymerase II inhibitor flavopiridol (FP; F3055; Sigma) or α-amanitin (A2263; Sigma) was added with a final concentration of 1 μM or 100 μg/ml for 1 h. The ATM inhibitor KU60019 (T2474; TagetMol) and PARP inhibitor Veliparib (T2591; TagetMol) were added with a final concentration of 10 μM for 1 h before micro-irradiation or for 3 days before homologous recombination repair assay. The DNA double-strand breaking reagent phleomycin (ant-zn-1p; InvivoGen) was treated with 50 ug/ml for 1 h. The nascent RNA transcript at transcribed active sites was detected with 5-ethynyl uridine (5-EU) following the manufacturer's protocol (Invitrogen). The doxycycline (D9891; Sigma), 4-hydroxytamoxifen (H6278; Sigma), and Shield1 ligand (#632189; Takara) were treated with 1 μM/ml for the indicated times to induce nascent transcription in U2OS 2-6-5 cell. To arrest cells at G1/S boundary or G2 phase, thymidine (T9250; Sigma) was treated with 2 mM.

### Antibodies and siRNAs

The information of antibodies or siRNAs used in this study was described in Supplementary Tables S1 and S2, respectively.

### Plasmids and mutagenesis

To generate pENTRY Donor vectors of human WSTF, EYA1, EYA3 and histone H2AX, PCR was amplified with individual ORF primer sets by human liver cDNA libraries. Then the clones into pENTRY were sequenced and transferred pcDNA-DEST47 (GFP C-terminal; Invitrogen), pcDNA-DEST53 (GFP N-terminal; Invitrogen) or pcDNA-DEST-FLAG vector using a Gateway LR Cloning system (Invitrogen). To generate WSTF and histone H2AX mutant plasmids, site-directed mutagenesis were carried out using the QuickChange site-directed mutagenesis kit (Stratagene) or by a classical PCR method using pENTRY clones. All mutation sites were validated by DNA sequencing analysis. FLAG-tagged RNA polymerase II (RNAPII) (#35175) and C-terminal deletion (ΔCTD) (#35176) constructs were purchased from Addgene. The detail information for plasmids or mutagenesis used in this study was summarized in Supplementary Tables S3 and S4, respectively.

### Immunofluorescence

Cells on glass bottom dishes were fixed with 2% (v/v) formaldehyde solution in PBS for 15 min at room temperature and washed once with PBS. Then, cells were blocked with 1% FBS/PBS for 20 min at room temperature. Primary antibodies were incubated with 1% FBS/0.5% Triton X-100 in PBS for overnight at 4°C. Cells were washed twice with 1% FBS/PBS, and then, incubated with secondary antibody for 1 h at room temperature. For RNase treatment, micro-irradiated cells were incubated for 1–2 h to allow RPA32 and RAD51 recruitment at laser stripes. Cells were permeabilized with 0.5% Triton X-100 for 5 min and treated with 3.5 mg/ml RNase A (Roche) in PBS or with 150 U/ml RNase H (TaKaRa) in 1× buffer (40 mM Tris-HCl, pH 7.7, 4 mM MgCl_2_, 1 mM DTT and 4% Glycerol) for 10 min at room temperature. Each well was mounted using Vectashield mounting medium with 4′,6-diamidino-2-phenylindole (DAPI) (Vector Labs). The signals were detected using a Nikon A1 confocal microscope.

### Immunoprecipitation and Western blot

HEK 293T cells were lysed with the modified NETN buffer (50 mM Tris, pH 8.0, 150 mM NaCl, 0.5% Nonidet P-40, 1 mM DTT, 50 U/ml MNase, 50 U/ml Benzonase) plus protease and phosphatase inhibitor cocktails (Roche). And cells were immunoprecipitated with 2 μg of anti-WSTF antibody (Santa Cruz Biotechnology) and anti-RNA polymerase II antibody (BioLegend), and added 25 μl of protein A-Sepharose beads (GE Healthcare Bio-Sciences). Cell lysates were incubated at 4°C for overnight. Interacting proteins were detected by Western blots. For western blot assay, samples were subjected to electrophoresis in 4–20% SDS-polyacrylamide gradient gels and immunoblotted with the indicated antibodies.

### Chromatin fractionation

Cells were first lysed with NETN buffer (50 mM Tris, pH 8.0, 150 mM NaCl, 0.5% Nonidet P-40, 1 mM DTT) plus protease and phosphatase inhibitor cocktails (Roche). And insoluble fractions were washed with NETN buffer three times. The fractions were lysed again with the modified NETN buffer (50 mM Tris, pH 7.4, 150 mM NaCl, 0.5% Nonidet P-40, 50 U/mL MNase, 50 U/ml Benzonase) plus protease and phosphatase inhibitor cocktails (Roche). Finally, the soluble fractions were used for western blots.

### Chromatin immunoprecipitation

The U2OS 2-6-5 reporter cells were treated with 4-hydroxytamoxifen and Shield1 ligand for 4 h to induce DNA double-strand breaks (DSBs) or released from induction by removal of 4-hydroxytamoxifen and Shield1 ligand for 4 h. Each of samples was used for chromatin immunoprecipitation as previously described (13).

### DSB-inducible transcription reporter assay

We carried out this experiment as previously described (13). Briefly, tandem tetracycline response elements (TREs) were integrated on chromosome 1p3.6 locus, which bind a doxycycline-inducible transactivator. The U2OS 2-6-5 cell, which can stably express YFP-MS2, was treated with doxycycline to induce nascent transcripts. Nascent active transcripts near to DSB sites can be visualized by accumulation of YFP-MS2 viral coat protein (see the Supplementary Figure S1G, H). mCherry-FokI signals at DSB sites were visualized by treatment with 4-hydroxytamoxifen and Shield1 for 4 h.

### Laser micro-irradiation

U2OS, HeLa, or HeLa H2AX KO cells were transfected with indicated GFP-fused or FLAG-fused constructs onto confocal glass bottom dishes (SPL) and were incubated with 10 μM 5-bromo-2′-deoxyuridine (BrdU, Calbiochem) for 30 h. Cells were subjected to mild condition of micro-irradiation for 1 s (16 lines/s) using wavelength 405 nm laser line at 37°C, 5% CO_2_. For detection signals at laser stripes, siRNA-, plasmids-transfected, or non-treated cells were incubated for the indicated times after micro-irradiation, and then fixed using 2% (v/v) formaldehyde solution in PBS for 15 min at room temperature. Each well was applied into immunocytochemistry as described in immunofluorescence section.

### Cell cycle arrest

To synchronize cells at G1/S boundary or G2 phase, we carried out double thymidine block assay. Briefly, first thymidine was treated into the cells for 18 h. At 9 h after washing out thymidine, secondary thymidine was treated for 16 h to arrest cells at G1/S boundary. To arrest at G2 phase, cells were released from secondary thymidine for 6 h.

### HR repair assay

An asynchronous U2OS based HR (DR-GFP) reporter cell was seeded into twelve-well plates, and next day, cell was treated with the indicated siRNAs. For G1 phase, cell was treated with the indicated siRNAs and was arrested with double thymidine. After 48 h, the asynchronous or G1-arrested cells were retreated with the siRNAs or siRNAs and the indicated plasmids along with I-SceI vector. Three days later, GFP signal arising from repair was measured by FACS analysis (BD Bioscience).

### Cell viability

U2OS, 293T, HeLa or HeLa H2AX KO cells were transfected with indicated siRNAs or siRNAs and indicated plasmids, and next day cells were seeded at a density of 500 cells in 6-cm dishes. The following day, cells were exposed to IR irradiation, as indicated, and cells were allowed to recover for 10 days. Colonies were fixed and stained with 0.5% crystal violet (C3886; Sigma), and then viability was expressed as the averages of data obtained from three independent experiments.

### Mass spectrometric analysis

HEK 293T cells were transfected with FLAG-WSTF or FLAG empty vector. At 48 h post-transfection, cells were immunoprecipitated with anti-FLAG-agarose (Sigma). The WSTF-interacting proteins were identified by mass spectrometry.

### Statistical analysis

Each experiment was independently repeated two or three times. Statistical analysis was performed using one-way ANOVA or unpaired Student's *t* test. Data are presented as means ± SEM. *P* value < 0.05 was considered statistically significant. Significance for each parameter of all data is indicated by asterisk (*P < 0.05; **P < 0.01; ***P < 0.005 versus the control).

## RESULTS

### Tyr142 phosphorylation of H2AX by WSTF is coupled to RNAPII-mediated active transcription in proliferating cells

**Figure 1.**
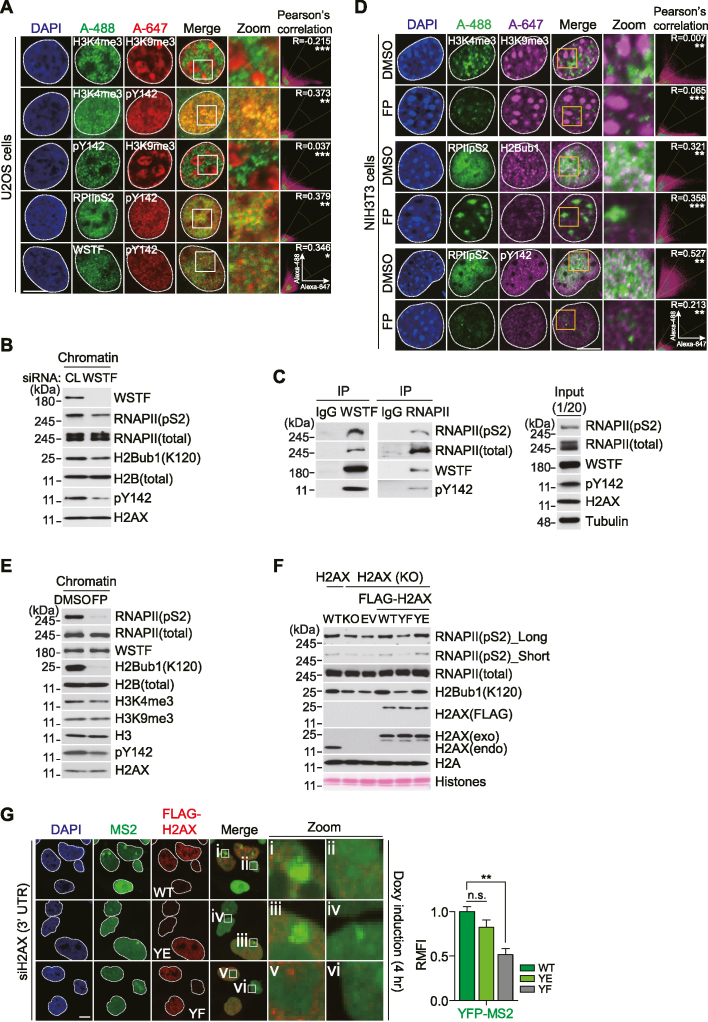
Tyr142 phosphorylation of H2AX by WSTF interacts with RNAPII and is coupled to RNAPII-mediated active transcription in proliferating cells. (**A**) Subcellular localizations of H2AX-pY142 (pY142), active RNA polymerase II (RPIIpS2), H3K4me3, H3K9me3, and WSTF. The proliferating U2OS cells were fixed with formaldehyde solution (2%) and immunostained with indicated antibodies. Scale bars, 10 μm. Data represent mean ± SEM of three independent experiments (n = 20). The ‘R’ means the Pearson's correlation coefficient. (**B**) Cells were transfected with control (CL) or WSTF siRNA for 48 h, and then isolated chromatin fractions were western-blotted with indicated antibodies. (**C**) An endogenous WSTF or RNA polymerase II (RNAPII) was immunoprecipitated with each antibody in proliferating 293T cells, and interactions among WSTF, RNAPII, RNAPIIpS2 and H2AX-pY142 (pY142) were detected by western blots. (**D**) Mouse fibroblast NIH3T3 cells were treated with dimethyl sulfoxide (DMSO) or flavopiridol (FP) for 4 h, and then cells were immunostained with indicated antibodies. Scale bars, 10 μm. Data represent mean ± SEM of two independent experiments (*n* = 30). The Pearson's correlation coefficient: R. (**E**) NIH3T3 fibroblasts were treated with an active RNA polymerase II inhibitor flavopiridol (FP) for 4 h as described in D, and then, isolated chromatin fractions were used for western blot with indicated antibodies. (**F**) Ectopically expressing FLAG-WT, FLAG-YF and FLAG-YE H2AX constructs were transfected into H2AX knock-out (KO) HeLa cells, and each of chromatin fractions was applied to western blot with indicated antibodies. (**G**) Ectopic FLAG-WT, FLAG-YE or FLAG-YF H2AX construct was transfected into the H2AX-depleted reporter cells, and cells were fixed and evaluated the accumulation of nascent transcript YFP-MS2 (MS2) after doxycycline (Doxy) induction for 4 h. Scale bars, 10 μm. Data represent mean ± SEM of two independent experiments (*n* = 50). n.s. means not significant.

To investigate the biological relevance of Tyr142 phosphorylation of H2AX by WSTF, we performed a mass-spectrometry analysis of recombinant WSTF (FLAG-WSTF) in proliferating 293T cells (Supplementary Figure S1A). FLAG-WSTF was tightly associated with the endogenous ISWI family subunits SNF2h, RSF1, and ACF1 (Supplementary Figure S1B), consistent with the results of previous studies (20–23). Notably, FLAG-WSTF also interacted with transcription activation factors, including the RNAPII subunits RPB1/RPB3 and the positive transcription elongation factors CDK12 and SPT5/SPT6 (Supplementary Figure S1B). To investigate its potential role in transcriptional regulation, we examined the association of H2AX-pY142 with active and inactive chromatin markers. Immunofluorescent staining of U2OS and HeLa cells revealed that endogenous H2AX-pY142 mainly correlated with markers of active transcription, including trimethylation of histone H3 at lysine 4 (H3K4me3) and RNAPIIpS2 (Figure 1A, Supplementary Figure S1C). By contrast, H2AX-pY142 did not associate with markers of inactive transcription, such as trimethylation of histone H3 at lysine 9 (H3K9me3) or lysine 27 (H3K27me3). In addition, siRNA-mediated depletion of WSTF reduced the level of chromatin-bound H2AX-pY142, as well as the levels of RNAPIIpS2 and RNAPIIpS2-coupled H2B Lys120 mono-ubiquitination (H2Bub1) (24) (Figure 1B). Next, to evaluate these associations, we performed an immunoprecipitation assay using an anti-WSTF or anti-RNAPII antibody. Immunoprecipitation with an anti-WSTF antibody co-precipitated H2AX-pY142, RNAPII and RNAPII phosphorylated at Ser2 in the C-terminal domain (RNAPIIpS2) (Figure 1C). Reciprocally, endogenous RNAPII was co-immunoprecipitated with WSTF and H2AX-pY142. These results suggest that WSTF-mediated formation of H2AX-pY142 is tightly associated with active transcription in proliferating cells.

Nuclear staining with 4′,6-diamidino-2-phenylindole (DAPI) can be used to detect regions of transcriptionally active (DAPI low) and transcriptionally inactive (DAPI high) chromatin, and mouse NIH3T3 fibroblasts are better suited to this method than human cells. Next, we treated NIH3T3 cells with the active RNAPII inhibitor flavopiridol (FP), which targets positive transcription elongation factor b, thereby inhibiting RNAPIIpS2 (24,25). Because cross-talk between H2Bub1 deposition and H3K4me3 has been reported previously (24), we utilized them as active RNAPIIpS2-coupled transcription marks. In NIH3T3 cells, a transcriptionally inactive region of chromatin with high DAPI staining colocalized with H3K9me3 immunostaining, whereas an active chromatin region with low DAPI staining colocalized with H3K4me3, RNAPIIpS2, H2Bub1 and H2AX-pY142 immunostaining. FP treatment reduced the levels of H3K4me3, RNAPIIpS2, and H2Bub1, but did not affect the level of H3K9me3 (Figure 1D). Concomitant with downregulation of RNAPIIpS2, H2Bub1, and H3K4me3, western blotting of chromatin-bound fractions revealed a reduction in the level of H2AX-pY142 following FP treatment, but the levels of RNAPII, WSTF, H2B, H3 and H3K9me3 were not affected (Figure 1E). In addition, the H2AX-pY142 level was markedly reduced in FP-treated U2OS cells (Supplementary Figure S1D).

Next, we examined whether H2AX-pY142 is important for RNAPII activity. Treatment of 293T cells with two different H2AX-specific siRNAs reduced the H2AX protein level markedly and resulted in low levels of chromatin-bound H2AX-pY142, RNAPIIpS2 and H2Bub1, but did not alter the levels of RNAPII, H2B or H2A (Supplementary Figure S1E). Furthermore, the levels of chromatin-bound RNAPIIpS2 and H2Bub1 were also reduced in H2AX knock-out (KO) cells (Supplementary Figure S1F). Next, we generated ectopic wild type (WT) H2AX (FLAG-WT), a Tyr142 phospho-mimetic mutant (FLAG-YE), and a Tyr142 phospho-dead mutant (FLAG-YF). Reconstitution of H2AX FLAG-WT or FLAG-YE into H2AX KO cells recovered the levels of RNAPIIpS2 and H2Bub1 at chromatin, while reconstitution of the FLAG-YF mutant did not (Figure 1F). Subsequently, we used an inducible transcription reporter system (13) to verify the cross-talk between H2AX-pY142 and transcription at active chromatin regions. Doxycycline treatment of a variant cell line expressing a YFP-tagged MS2 viral coat protein (YFP-MS2) and a reporter transcript resulted in co-accumulation of RNAPIIpS2 with YFP-MS2 after 4 h (Supplementary Figure S1G, H). Depletion of H2AX using a siRNA reduced the enrichment of YFP-MS2 at the reporter locus after doxycycline induction, and reconstitution of WT H2AX or a Y142E mutant into the H2AX-depleted cells recovered the YFP-MS2 signal, but did not recover with Y142F mutant (Figure 1G). Overall, these findings suggest that the WSTF-mediated H2AX-pY142 level is tightly linked to RNAPII-dependent transcription activation in proliferating cells.

### The WSTF-H2AX-pY142-RNAPII axis is disrupted during the DDR

**Figure 2.**
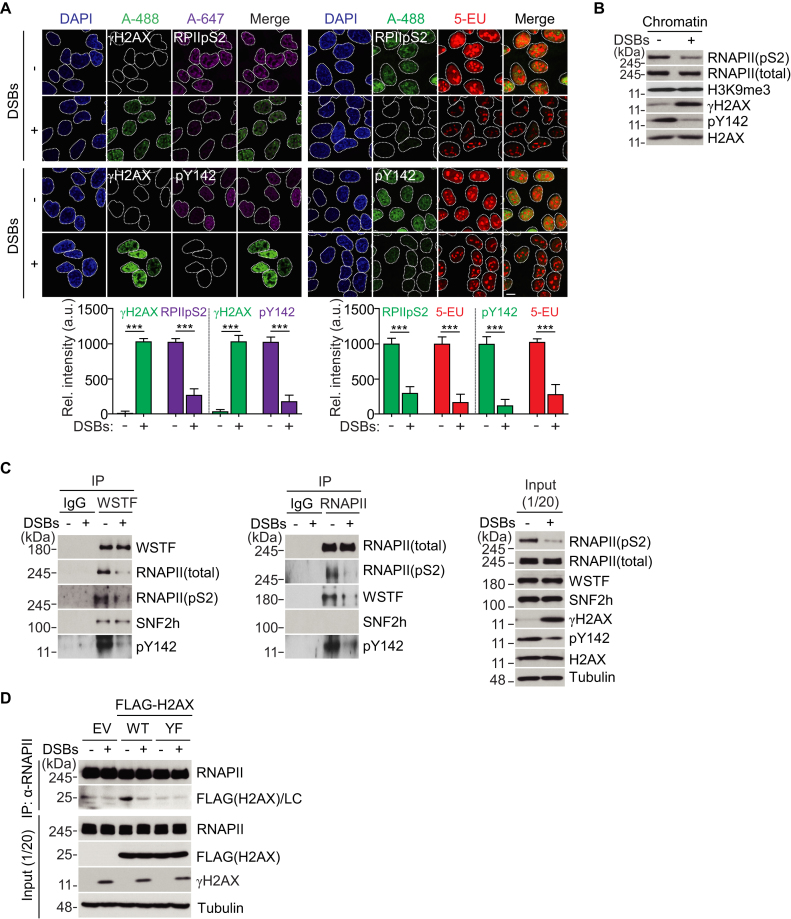
The WSTF-H2AX-pY142-RNAPII axis is disrupted during the DDR. (**A**) An intensity of γH2AX, active RNA polymerase II (RPIIpS2), H2AX-pY142 (pY142) and 5-EU without or with DNA damage. Cells were treated (+) or non-treated (–) with phleomycin (50 μg/ml) for 1 h. Cells were fixed and immunostained with indicated antibodies. Scale bars, 10 μm. Data represent mean ± SEM of three independent experiments. (**B**) Cells were treated with phleomycin as described in A, and then isolated chromatin fractions were western-blotted with indicated antibodies. (**C**) An endogenous WSTF or RNA polymerase II (RNAPII) was immunoprecipitated with each antibody in 293T cells after treatment with or without phleomycin for 1 h, and interacting proteins were detected by western blot. (**D**) Ectopically expressing FLAG-WT and FLAG-YF H2AX constructs were transfected into 293T cells, and an endogenous RNA polymerase II (RNAPII) was immunoprecipitated with anti-RNAPII antibody after treatment with or without phleomycin for 1 h, and interacting H2AX proteins were detected by western blot.

Because it was reported that the level of pre-existing H2AX-pY142 is abolished upon DNA damage (7), we hypothesized that the tightly associated WSTF-H2AX-pY142-RNAPII axis might be disrupted under DNA damage conditions. The H2AX-pY142 and RNAPIIpS2 signals were diminished at DNA lesions and damaged chromatin, and 5-ethynyluridine (5-EU)-detectable global RNA transcripts were inhibited upon DNA damage (Figure 2A, B). To evaluate this hypothesis, we again performed an immunoprecipitation assay using an endogenous WSTF or RNAPII antibody under non-damaged or damaged conditions. As expected, in normal cells, endogenous WSTF was tightly associated with the positive control binding partner SNF2h, as well as RNAPII (total and pS2) and H2AX-pY142. Reciprocally, endogenous RNAPII also interacted with WSTF and H2AX-pY142, but not SNF2h. Upon DNA damage, the total levels of the RNAPII, WSTF, and H2AX proteins were not changed, but the WSTF-H2AX-pY142-RNAPII axis was disrupted (Figure 2C). Immunofluorescence staining and western blotting revealed that H2AX-pY142 and RNAPIIpS2 were downregulated in response to DNA damage (Figure 2A, B), which may weaken the interaction between H2AX-pY142 and RNAPII. Thus, we reconstituted H2AX FLAG-WT or FLAG-YF into H2AX KO cells. In immunoprecipitation experiments, endogenous RNAPII interacted with ectopic FLAG-WT H2AX, but this interaction was disrupted under DNA damage conditions (Figure 2D). By contrast, the H2AX Tyr142 phospho-dead mutant (FLAG-YF) did not interact with RNAPII under non-damaged or damaged conditions. These data indicate that pre-existing Tyr142 phosphorylation of H2AX by WSTF is connected to RNAPII-mediated active transcription regulation under basal conditions, but this association is disrupted during the DDR.

### Tyr142 dephosphorylation of H2AX by EYAs is indispensable for DNA damage-induced transcriptional silencing

**Figure 3.**
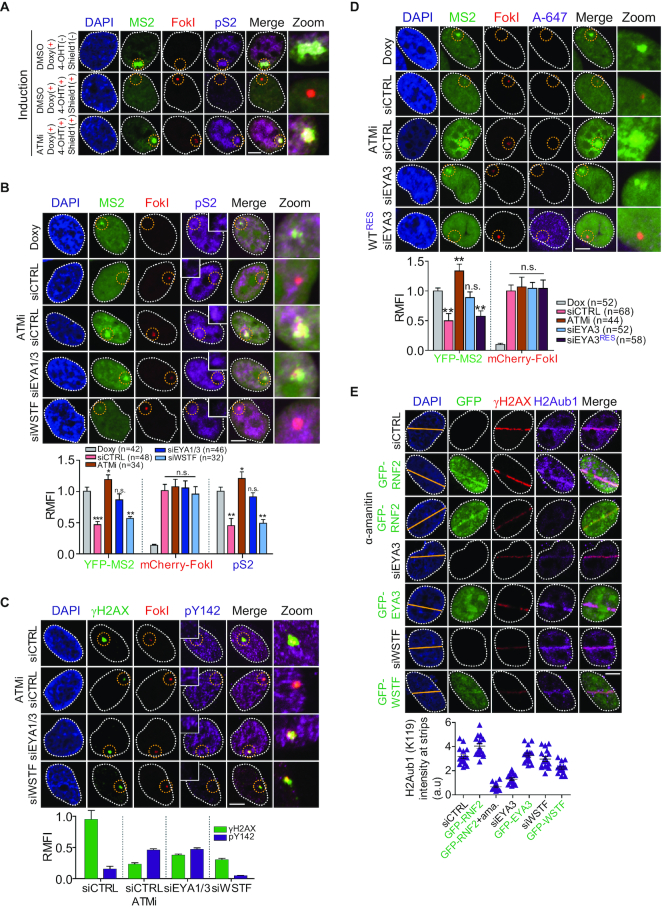
H2AX-pY142 dephosphorylation by ATM-dependent EYAs is required for transcriptional silencing at transcribed active DSB sites. (**A**) An enrichment of viral coat protein YFP-MS2 at transcribed active sites is reduced upon DNA damage. An inducible transcription reporter cell was treated with doxycycline (Doxy) or Doxy/4-OHT/Shield1 for 4 h and ATM inhibitor (ATMi) was used for DSB site-specific transcription silencing inhibitor, which was treated at 1 h before adding of Doxy/4-OHT/Shield1. Cells were fixed and immunostained with anti-RNA polymerse II phospho-Ser2 antibody (pS2). Scale bars, 10 μm. (**B–D**) The reporter cells were transfected with the indicated siRNAs or siRNAs and plasmid. At 48 h post-transfection, cells were induced as described in A. Cells were fixed and immunostained with anti-RNA polymerse II phospho-Ser2 (pS2) (B), anti-pY142 (C), and anti-FLAG (WT^RES^siEYA3) (D) antibodies. Scale bars, 10 μm. Data represent mean ± SEM of three independent experiments. n.s. means not significant. (**E**) U2OS cells were transfected with the indicated siRNAs or GFP-fused constructs. At 48 h post-transfection, cells were fixed at 10 min post-microirradiation and immunostained with indicated antibodies. An α-amanitin was used for transcriptional inhibition. Scale bars, 10 μm. Data represent mean ± SEM of two independent experiments.

An ablation of H2AX-pY142 by ATM-dependent EYA1/3 phosphatases is critical for γH2AX-dependent DNA damage signals and repair (8). In addition, given its strong link to the active transcription factor RNAPII was abolished in response to DNA damage, we hypothesized that removal of pre-existing H2AX-pY142 by EYA phosphatases may involve local transcriptional regulation at DNA damage sites. To investigate this hypothesis, we used a locally inducible transcription reporter system at DSBs (13) (Supplementary Figure S2A). In U2OS cells stably expressing mCherry-FokI endonuclease, treatment with 4-hydroxytamoxifen and Shield1 ligand to promote FokI expression and translocation to DNA double-strand break sites (DSBs) induced the activation and accumulation of DNA damage signaling cascades and repair factors at DSBs in time-dependent manner (Supplementary Figure S2B–E). As expected, in the absence of FokI endonuclease induction, the nascent YFP-MS2 transcript and RNAPIIpS2 were accumulated at or near to DSBs upon doxycycline addition, as described in the supplementary figures S1G and S1H. However, these signals were eliminated by promoting mCherry-FokI expression by treatment with 4-hydroxytamoxifen and Shield1 ligand, and were induced by inhibition of ATM activity (13) (Figure 3A, Supplementary Figure S2F). We then used the inducible reporter system to determine whether targeting of H2AX-pY142 by WSTF or EYAs affects the transcription status in the presence or absence of DNA damage. Induction of mCherry-FokI abolished the YFP-MS2, active RNAPII (pS2), and H2AX-pY142 signals at DSBs in reporter cells transfected with a control or WSTF-specific siRNA. By contrast, inhibition of ATM activity and depletion of the ATM targets EYA1 and EYA3 affected transcriptional activation at DSBs (Figure 3B, C, Supplementary Figure S2G). However, reconstitution of an EYA3-targeting siRNA-resistant wild type (WT^RES^siEYA3) caused transcriptional silencing at DSBs in a similar manner to that seen in the controls (Figure 3D). In mammalian cells, targeting of the mono-ubiquitination of histone H2A at Lys119 (H2Aub1) by the E3 ubiquitin ligase RNF2/RING1B is a marker of transcriptional repression at DNA lesions (26). Thus, we determined whether dephosphorylation of H2AX-pY142 is required for transcriptional silencing at damaged chromatin. In agreement with the DSB reporter system results, high levels of RNF2 and EYA3 enriched the level of H2Aub1 at laser stripes in irradiated U2OS cells, but the formation of this modification was hindered by treatment of the cells with the RNAPII inhibitor α-amanitin or by knock-down of EYA3 (Figure 3E). As expected, overexpression or depletion of WSTF did not affect local transcription repression at DSBs. Taken together, these results indicate that dephosphorylation of H2AX-pY142 by ATM-dependent EYAs plays a crucial role in local transcriptional silencing at DSBs in a WSTF-independent manner.

### Active RNAPII is required for HR repair at the G1 phase

**Figure 4.**
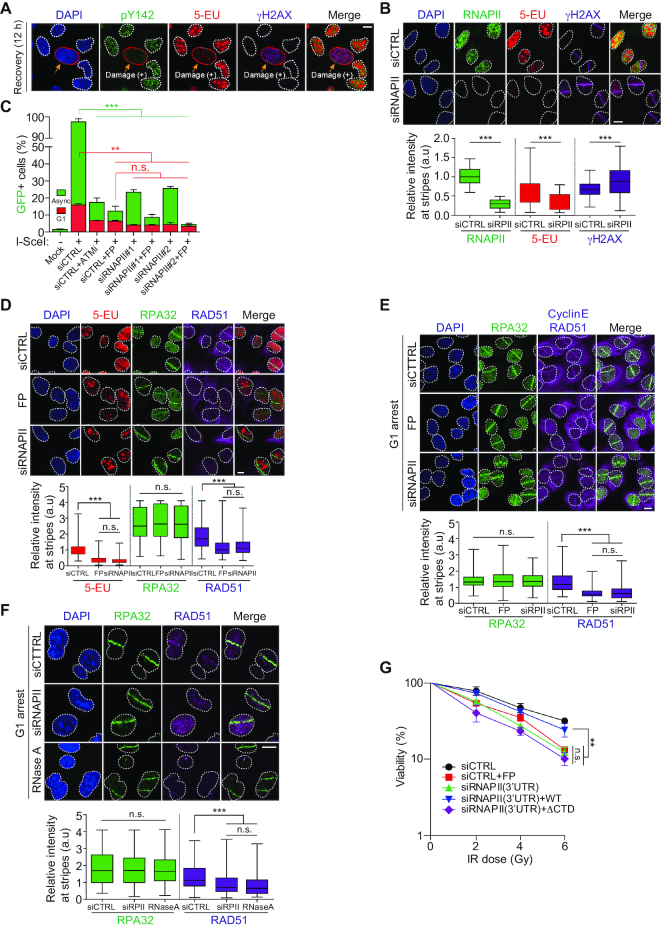
Active RNAPII is required for HR repair at the G1 phase. (**A**) To recover after DNA damage breaks, U2OS cells were treated with phleomycin (10 μg/ml) for 1 h, and then phleomycin-removed cells were maintained into new medium for 12 h. Fixed cells were immunostained with anti-pY142 and anti-γH2AX antibodies as well as 5-EU. Scale bars, 10 μm. (**B**) U2OS cells were transfected with control siRNA or RNAPII-targeting siRNA for 48 h. Micro-irradiated cells were immunostained with anti-RNAPII and anti-γH2AX antibodies as well as 5-EU after 2 h. Scale bars, 10 μm. Data represent mean ± SEM of more than 20 cells. (**C**) Comparative analysis of HR repair by depletion or inhibition of RNAPII in asynchronous or G1-arrested HR reporter cells. ATM inhibitor (ATMi) or flavopiridol (FP) was used for efficiency of HR repair or of transcription inhibition, respectively. Data represent mean ± SEM of three independent experiments. n.s. means not significant. (**D**) U2OS cells were transfected with control siRNA or RNAPII-targeting siRNA for 48 h. Micro-irradiated cells were immunostained with indicated antibodies and 5-EU after 2 h. Flavopiridol (FP) was treated before 1 h microirradiation. Scale bars, 10 μm. Data represent mean ± SEM of three independent experiments. n.s. means not significant. (**E** and **F**) HeLa cells were transfected with RNAPII-targeting siRNA and arrested at G1 phase by double thymidine block. Flavopiridol (FP) was treated before 1 h microirradiation (E) or RNase A was treated after 2 h microirradiation (F). Fixed cells at 2 h post-microirradiation were immunostained with indicated antibodies. An anti-Cyclin E antibody was used for G1-specific. Scale bars, 10 μm. Data represent mean ± SEM of three independent experiments. n.s. means not significant. (**G**) 293T cells were transfected with RNAPII 3′-UTR-targeting siRNA (3′UTR) or siRNAPII (3′UTR) and ectopic FLAG-RNAPII constructs (WT or ΔCTD) for 48 h, then cells were irradiated with IR at the indicated dose. The viability was performed by colony forming assay. Data represent mean ± SEM of three independent experiments.

Recently, it was suggested that active RNAPII-mediated RNA transcripts control efficient DNA damage repair during cell cycle progression (17–19,27). We also found that the recovery of the level of H2AX-pY142 relative to that of γH2AX was tightly coupled with 5-EU-detectable RNA transcripts after DNA damage, while impaired regions of DNA or micro-irradiated regions with depleted RNAPII retained γH2AX signals and diminished 5-EU signals (Figure 4A, B). These findings suggest that the axis of H2AX-pY142 formation and RNAPII-mediated active RNA transcription may be required for ensuring proper DNA damage repair. Thus, we focused on determining whether RNAPII-mediated active transcription is required for DNA damage repair during cell cycle progression, using HR reporter U2OS cells (28,29). HR repair was inhibited by depletion of RNAPII in these asynchronous cells, and a synergistic effect was seen following treatment with FP (Figure 4C, Supplementary Figure S3A). In G1 phase, the portion of total HR repair was markedly less than in asynchronous cells (15% versus 100%). The statistics of inhibition of RNAPII activity, depletion of RNAPII, as well as combination of RNAPII inhibition and depletion did not show any significance in G1-specific HR repair, but not in asynchronous HR repair (Figure 4C). These data indicate that RNAPII-mediated HR efficiency is more convincing in G1 phase, whereas RNAPII-mediated HR might be more complicated during S/G2 phase. To identify the repair factors involved in this pathway, we immunostained control, RNAPII-depleted, or FP-treated micro-irradiated U2OS cells with HR repair-specific antibodies. Recruitment of the HR strand invasion factor RAD51 and 5-EU signals at the laser stripes were reduced in RNAPII-depleted and FP-treated cells, but recruitment of RPA32, another HR DNA end resection factor, was not affected by RNAPII depletion and FP treatment (Figure 4D, Supplementary Figure S3B). HR repair mainly occurs in the S/G2 phase because of the presence of sister chromatids as a donor template. Recently, several reports suggested that HR also occurs in the G1 phase and, in the absence of sister chromatids, uses damage-induced active RNA transcripts as HR donor templates (19,30). Given that recruitment of RAD51 and 5-EU at laser stripes was reduced under RNAPII-depleted conditions, but recruitment of RPA32 was not, we hypothesized that RAD51 recruitment might be controlled in a cell cycle-dependent manner. To investigate this hypothesis, cells were arrested in the G1 phase by the double-thymidine block (DTB) method or S/G2 phase after 6 h release from the DTB. Surprisingly, downregulation or FP-mediated inhibition of RNAPII repressed RAD51 recruitment in the G1 phase rather than the S/G2 phase, whereas RPA32 was properly accumulated at the laser stripes in both phases, regardless of RNAPII inhibition or downregulation (Figure 4E, Supplementary Figure S3C). Subsequently, we examined the dependency of RAD51 recruitment at laser stripes in the G1 phase on RNAPII-mediated active RNA transcripts. Following RNase A-mediated destruction of the RNA template, RAD51 was no longer accumulated at the laser stripes (Figure 4F). At transcribed sites, single-stranded RNA (ssRNA) forms R-loops and re-hybridizes with ssDNA. In this case, RNase A is not able to degrade template RNA. Thus, we examined again using RNase H on the recruitment of RPA32 and RAD51 at the laser stripes. As expected, RPA32 was properly recruited at DNA lesions in U2OS cells treated with RNase H, whereas RAD51 was not recruited at the sites (Supplementary Figure S3D). Furthermore, a clonogenic assay showed that RNAPII-mediated active RNA transcripts supported cell viability after DNA damage. The FP-mediated inhibition of RNAPII and reduction of RNAPII levels by siRNA reduced the cell viability. Reconstitution of RNAPII WT, but not CTD-truncated RNAPII mutant (ΔCTD), restored the cell viability (Figure 4G). These data indicate that active RNAPII-mediated RNA transcripts are necessary for RAD51 recruitment for HR repair during the G1 phase.

### Tyr142 rephosphorylation of H2AX is important for RAD51 loading during the G1 phase

**Figure 5.**
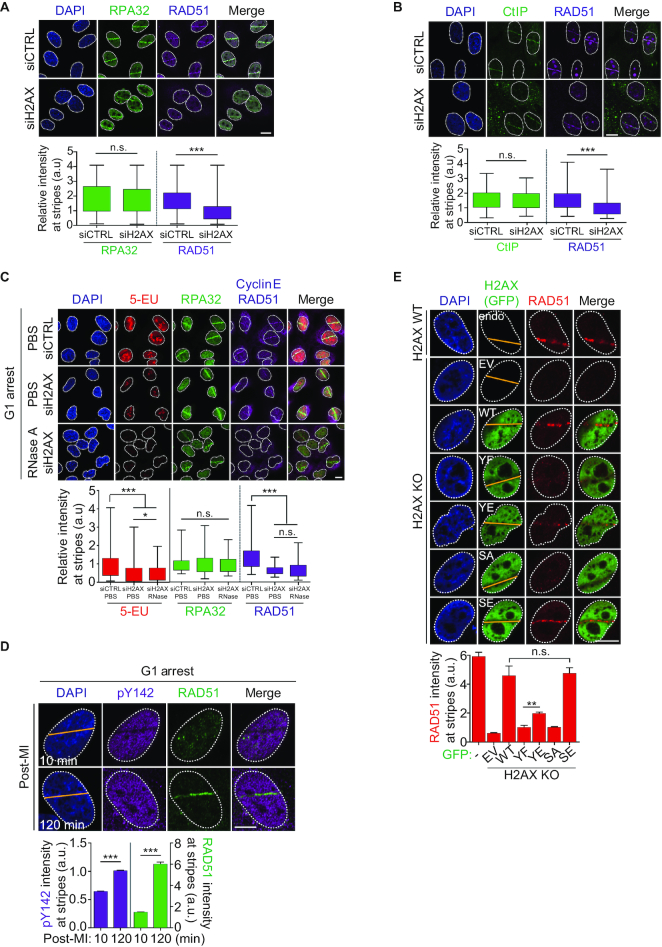
Tyr142 rephosphorylation of H2AX is important for RAD51 loading during the G1 phase. (**A** and **B**) U2OS cells were transfected with control siRNA or H2AX-targeting siRNA for 48 h. At 2 h post-microirradiation, fixed cells were immunostained with anti-RPA32 and anti-RAD51 antibodies (A), or anti-CtIP and RAD51 antibodies (B). Scale bars, 10 μm. Quantitative analysis for the fluorescence intensity of signals at laser stripes. Data represent mean ± SEM of three independent experiments. n.s. means not significant. (**C**) HeLa cells were transfected with H2AX-targeting siRNA and arrested at G1 phase by double thymidine block. RNase A was treated after 2 h post-microirradiation. Fixed cells were immunostained with indicated antibodies and 5-EU. An anti-Cyclin E antibody was used for detecting G1-arrested cells. Scale bars, 10 μm. Data represent mean ± SEM of three independent experiments. n.s. means not significant. (**D**) The recruitment of endogenous H2AX-pY142 and RAD51 at the laser stripes was detected by immunostaining with anti-pY142 and anti-RAD51 antibodies at 10 min or at 2 h post-microirradiation in G1-arrested U2OS cells. Scale bars, 10 μm. Data represent mean ± SEM of three independent experiments. (**E**) GFP-fused H2AX constructs were introduced into H2AX KO cells. Micro-irradiated cells were fixed after 2 h. The recruitment of RAD51 at laser stripes was detected with anti-RAD51 antibody. An empty vector (EV) into H2AX KO cell was used as a negative control. Scale bars, 10 μm. Data represent mean ± SEM of more than 20 individual cells. n.s. means not significant.

Next, we addressed whether rephosphorylation of H2AX-Y142 is related to RNAPII-mediated HR repair in the G1 phase. To this end, we immunostained U2OS cells with anti-RAD51 and anti-RPA32 antibodies at 2 h post-laser micro-irradiation. The RAD51 signal at the laser stripes was not rescued following depletion of H2AX, whereas RPA32 proteins were accumulated at the stripes in H2AX-depleted cells at a similar level to that seen in the controls (Figure 5A, Supplementary Figure S4A). Broken DNA should be resected by the exonuclease CtIP before loading of RPA32, which binds and stabilizes single-stranded DNA (ssDNA) intermediates for HR repair (31,32). Next, we tested again whether CtIP recruitment at DNA lesions is dependent on H2AX expression. We found that CtIP recruitment was not affected by knock-down of H2AX (Figure 5B). To investigate whether RNA templates are required for HR repair in G1 phase, WT and H2AX-depleted cells were synchronized at G1 phase by the DTB and micro-irradiated WT and H2AX-depleted cells were co-stained with 5-EU and antibodies against RPA32, Cyclin E, and RAD51. Recruitment of RAD51 at the laser stripes was inefficient in H2AX-depleted cells, and the combination of H2AX depletion and disruption of RNA transcripts by RNase A also perturbed RAD51 loading (Figure 5C).

An importance of H2AX on RAD51 loading appeared to be G1 phase-specific because Rad51 loading was not affected in cells co-stained with cyclin B1 that accumulates during S/G2 phase (Supplementary Figure S4A, B). Next, to investigate whether rephosphorylation of H2AX-Y142 is important for RAD51 loading at the laser stripes during the G1 phase, micro-irradiated cells were immunostained with anti-pY142 and anti-RAD51 antibodies after micro-irradiation. Initially, an endogenous H2AX-pY142 signal was dissociated from the laser stripes where RAD51 was not recruited. However, H2AX-pY142 was filled at the laser stripes and RAD51 was accumulated at the lesions after 2 h post-microirradiation (Figure 5D). In addition, we found that reconstitution of YE H2AX mutant (mimetic form of H2AX-pY142), but not phosphorylation defective YF H2AX mutant, in H2AX knockout cells partially but consistently rescued RAD51 loading at DNA lesions (Figure 5E, Supplementary Figure S4C), suggesting that rephosphorylation of H2AX-Y142 is important for RAD51 loading in G1 phase. Reconstitution of H2AX-SE mutant (mimetic form of γH2AX) was as efficient as WT in RAD51 loading because it seemed that the signal of γH2AX is crucial for RAD51 through S/G2 phase. Collectively, these results indicate that rephosphorylation at Tyr142 of H2AX is likely to augment HR repair by recruitment of RAD51 at DSBs in the G1 phase.

### *De novo* phosphorylation of H2AX-Y142 by WSTF facilitates RNAPII-mediated TC-HR repair at the G1 phase

**Figure 6.**
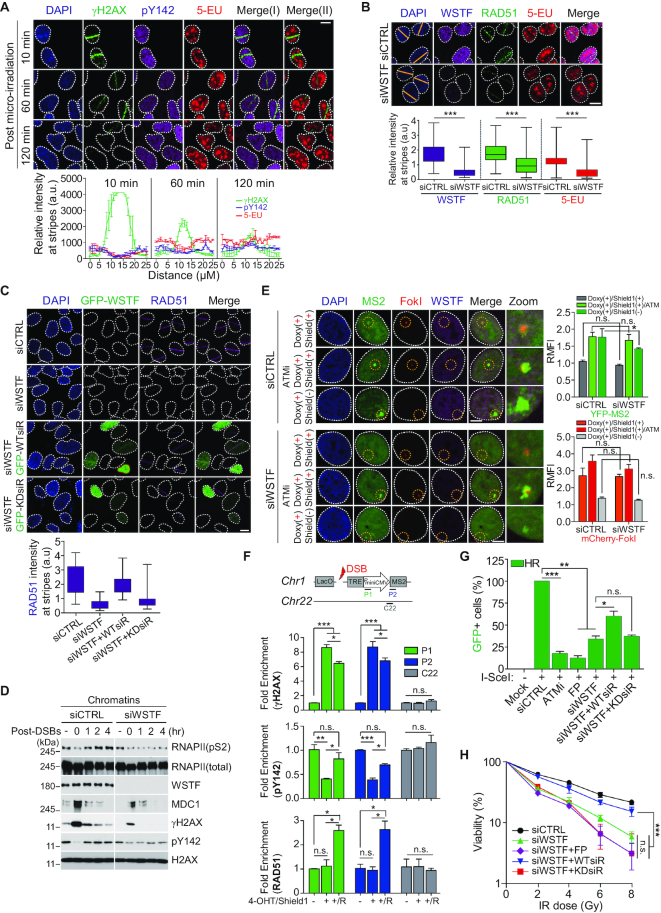
*De novo* phosphorylation of H2AX-Y142 by WSTF facilitates RNAPII-mediated TC-HR repair at the G1 phase. (**A**) U2OS cells were immunostained with γH2AX and pY142 antibodies as well as 5-EU at indicated time points after microirradiation (upper panels). Scale bars, 10 μm. Quantitative analysis for the fluorescence intensity of signals at laser stripes (lower panels). Data represent mean ± SEM of more than 20 individual cells. (**B**) U2OS cells were transfected with control or WSTF siRNA and arrested at G1 phase by using double thymidine block. At 48 h post-transfection, cells were microirradiated, and at 2 h later fixed cells were immunostained with indicated antibodies and 5-EU. Scale bars, 10 μm. Data represent mean ± SEM of three independent experiments. (**C**) U2OS cells were transfected with WSTF-targeting siRNA or WSTF siRNA and WSTF siRNA-resistant GFP-WSTF wild type (GFP-WTsiR) or kinase-dead mutant (GFP-KDsiR) and arrested at G1 phase with double thymidine block. At 48 h post-transfection, cells were microirradiated and fixed at 2 h post-microirradiation. Fixed cells were immunostained with anti-RAD51 antibody. Scale bars, 10 μm. Quantitative analysis for recruitment of RAD51 at laser stripes. Data represent mean ± SEM of >20 individual cells. (**D**) U2OS cells were transfected with control siRNA or WSTF siRNA. At 48 h post-transfection, cells were treated with phleomycin (50 μg/ml) for 1 h. After washing-out, cells were harvested at indicated time points. The chromatin fractions were used for Western blots. (**E**) The reporter cells were transfected with the indicated siRNAs. At 48 h post-transfection, cells were induced by adding with doxycycline, 4-hydroxytamoxifen and Shield1 ligand or removing of Shield1 ligand. ATM inhibitor was used for transcription activation at DSB sites. Cells were fixed and immunostained with anti-WSTF antibody. Scale bars, 10 μm. Data represent mean ± SEM of three independent experiments. n.s. means not significant. (**F**) The kinetics of γH2AX, pY142 and RAD51 enrichment at DSB site. Indicated antibodies were used for chromatin immunoprecipitation assay. Data represent mean ± SEM of two independent experiments. n.s. means not significant. (**G**) Comparative analysis of HR repair by reconstitution of WSTF constructs (WTsiR and KDsiR) into WSTF knock-downed HR reporter cells. Data represent mean ± SEM of three independent experiments. n.s. means not significant. (**H**) 293T cells were transfected with WSTF-targeting siRNA (siWSTF) or siWSTF and ectopic FLAG-WSTF constructs (WTsiR or KDsiR) for 48 h, then cells were irradiated with IR at the indicated dose. The viability was performed by colony forming assay. Data represent mean ± SEM of three independent experiments. n.s. means not significant.

Next, we investigated whether H2AX-pY142-targeting WSTF is recruited to DNA lesions. As previously reported (29,33,34), the IWSI family subunits SNF2h and RSF1 were recruited to DNA lesions (Supplementary Figure S5A,B). Exogenous GFP-fused WSTF (GFP-WSTF) and endogenous WSTF colocalized with DSB-inducible mCherry-FokI and γH2AX signals at micro-irradiated damage sites, respectively (Supplementary Figure S5A, B). In addition, an abrogated H2AX-pY142 signal was rescued and WSTF accumulated after micro-irradiation in a time-dependent manner, while γH2AX signals gradually decreased (Supplementary Figure S5C, D). Generally, it is believed that a histone variant H2AX is phosphorylated in its pre-existing location in chromatin upon DNA damage, not that H2AX is recruited to damage sites or evicted from damage sites. Thus, we evaluated that the signals of pY142, γH2AX, and H2AX at DNA lesions after laser micro-irradiation. Expectedly, signals of pY142 and γH2AX showed the invert relationship, whereas total H2AX did not alter at damaged or undamaged regions (Supplementary Figure S5E). Next, to determine whether the axis of WSTF-mediated H2AX-pY142 and RNAPII-mediated RNA transcripts relies on DNA damage repair, we examined the kinetics of the H2AX-pY142, 5-EU, and γH2AX signals after micro-irradiation of U2OS cells. The initially depleted H2AX-pY142 and 5-EU signals were restored at DNA lesions in a time-dependent manner, and were almost fully recovered 2 h after micro-irradiation (Figure 6A). On the other hand, the early amplified γH2AX signals degenerated after DNA damage repair. Next, we examined whether WSTF-mediated H2AX-Y142 *de novo* phosphorylation is required for TC-HR repair at the G1 phase. RAD51 and 5-EU were not recovered at the laser stripes at 2 h post-micro-irradiation in WSTF-depleted G1 cells (Figure 6B, Supplementary Figure S5F). Furthermore, the decrement of RAD51 recruitment at laser stripes in WSTF-depleted cells was rescued by the addition of siRNA-resistant GFP-tagged WT WSTF (GFP-WT*siR*), while a kinase-dead mutant WSTF (GFP-KDsiR) was dispensable for RAD51 recruitment (Figure 6C, Supplementary Figure S5G). In addition, GFP-tagged WT WSTF was accumulated at DNA lesions, but the kinase-dead mutant was not (Supplementary Figure S5H). Next, we isolated the chromatin fractions at different time points after release of control or WSTF-depleted U2OS cells from the DNA-damaging agent phleomycin. In control cells, initially amplified γH2AX and MDC1 signals were markedly decreased and H2AX-pY142 and RNAPIIpS2 signals were recovered gradually after DNA damage, but this recovery did not occur in WSTF-depleted cells (Figure 6D). As expected, in the presence of 4-hydroxytamoxifen, Shield1 ligand and FokI endonuclease induction, the nascent YFP-MS2 transcript was eliminated from DSBs, while this signal was accumulated at DSBs by inhibition of ATM activity or removing of Shield1 in a control, but not with removing of Shield1 in WSTF-depleted U2OS stable cells (Figure 6E). To conclusively demonstrate H2AX-pY142-mediated RAD51 recruitment at DSB site (DSBs), we performed the kinetics of H2AX-pY142, γH2AX, and RAD51 enrichment at DSB site by using chromatin immunoprecipitation (ChIP) assay. γH2AX signal enriched at DSBs was decreased after releasing DNA breaks, whereas H2AX-pY142 signal was rescued at a similar level to normal and RAD51 was accumulated at DSBs after releasing DNA breaks, indicating that recovery of pY142-H2AX and recruitment of RAD51 at DSBs coordinate TC-HR repair (Figure 6F). Finally, we focused on efficient HR repair. The siRNA-resistant WT WSTF (WTsiR) recovered HR repair efficiently, while the kinase-dead mutant WSTF (KDsiR) did not (Figure 6G). A clonogenic assay of U2OS cells exposed to ionizing radiation revealed that reconstitution of ectopic WT WSTF into WSTF-depleted cells rescued cell survival, but the kinase-dead WSTF mutant did not. In addition, FP-mediated inhibition of transcription did not rescue cell survival (Figure 6H). Collectively, these results demonstrate that *de novo* phosphorylation of H2AX at Tyr142, mediated via translocation of WSTF to DNA lesions, is necessary for RNAPII-mediated TC-HR repair by RAD51 loading in G1-specific.

## DISCUSSION

H2AX is abundantly accumulated at transcribed active regions and is present at lower levels at heterochromatin and near centromeres (6,35). Depletion of γH2AX is likely to cause transcriptional maintenance on active genes (6). Indeed, the presence of γH2AX and its target kinases blocks progressive transcription on active chromatin regions (12,36). Spreading of γH2AX on transcribed genes may protect and maintain proper transcriptional regulation.

Here, we found that Tyr142 phosphorylation of H2AX by WSTF interacts with RNAPII and is enriched with active histone markers at transcriptionally active regions in non-damaged conditions. However, this signal is initially restrained by EYA phosphatase activity in an ATM-dependent manner following DNA damage. As WSTF antagonists, the EYA family of proteins, encoded by the *EYA1* and *EYA3* genes, act as transcription cofactors that play a role in the retinal development system, and have tyrosine-specific phosphatase activity in the DDR (8,37). Notably, EYA1/3 is unlikely to target Tyr142 of H2AX under basal conditions. On the other hand, ATM-dependent EYA activity is specific for removal of sustained Tyr142 phosphorylation by WSTF in the DDR. In addition, it seems that the activity of EYAs is much stronger than that of WSTF in the conflict of these proteins at DSB sites, as indicated by the disappearance of the Tyr142 phosphorylation signals at early stages after chromatin damage (Figure 6A, Supplementary Figure 5C, E). Recently, it was reported that transcriptional silencing at DSB sites relies on ATM kinase activity and histone repressive marks, which are established by chromatin writers and readers at damaged chromatins (9–13). We found that ATM-mediated EYA1/3 activation is important for H2AX-pY142-specific dephosphorylation through γH2AX docking, which regulates local transcription silencing at the damaged locus, whereas WSTF is dispensable for transcriptional repression in the early DDR (Figure 3). Thus, it is reasonable to hypothesize that an initial ablation of pre-existing H2AX-pY142 promotes γH2AX amplification and transcriptional silencing at transcribed active DSB sites.

DSBs are mainly repaired by either HR or c-NHEJ, and these processes are controlled during cell cycle progression. In principle, HR repair occurs in the S/G2 phase and is error-free because of the necessity of using the sister chromatid as a template. By contrast, c-NHEJ is the dominant pathway in G1 due to the absence of sister chromatids, which can cause cancer and genetic disorders from error-prone repair through small deletions or insertions at break junction sites. Accordingly, error-free repair mechanisms are essential for the proper correction of genetic information. One of the issues at damaged active genes is how to regulate transcription status and repair to maintain genome integrity. Notably, c-NHEJ also enhances error-free repair of transcribed genes via cross-talk between RNAPII and c-NHEJ factors, but this process does not occur on non-transcribed genes, where RNAPII-dependent nascent RNA can serve as a template for accurate repair (17). Furthermore, DNA damage-responsive RNAs (DDRNAs) control DDR activation and repair via interaction between the MRN complex and RNAPII to induce 53BP1 foci formation in the early DDR (18). Meanwhile, limited evidence supports that DNA damage on transcriptionally active genes is required for nascent RNA formation and RNAPII activity to specifically recruit HR factors for TC-HR repair in a cell cycle-dependent manner (16,19).

**Figure 7.**
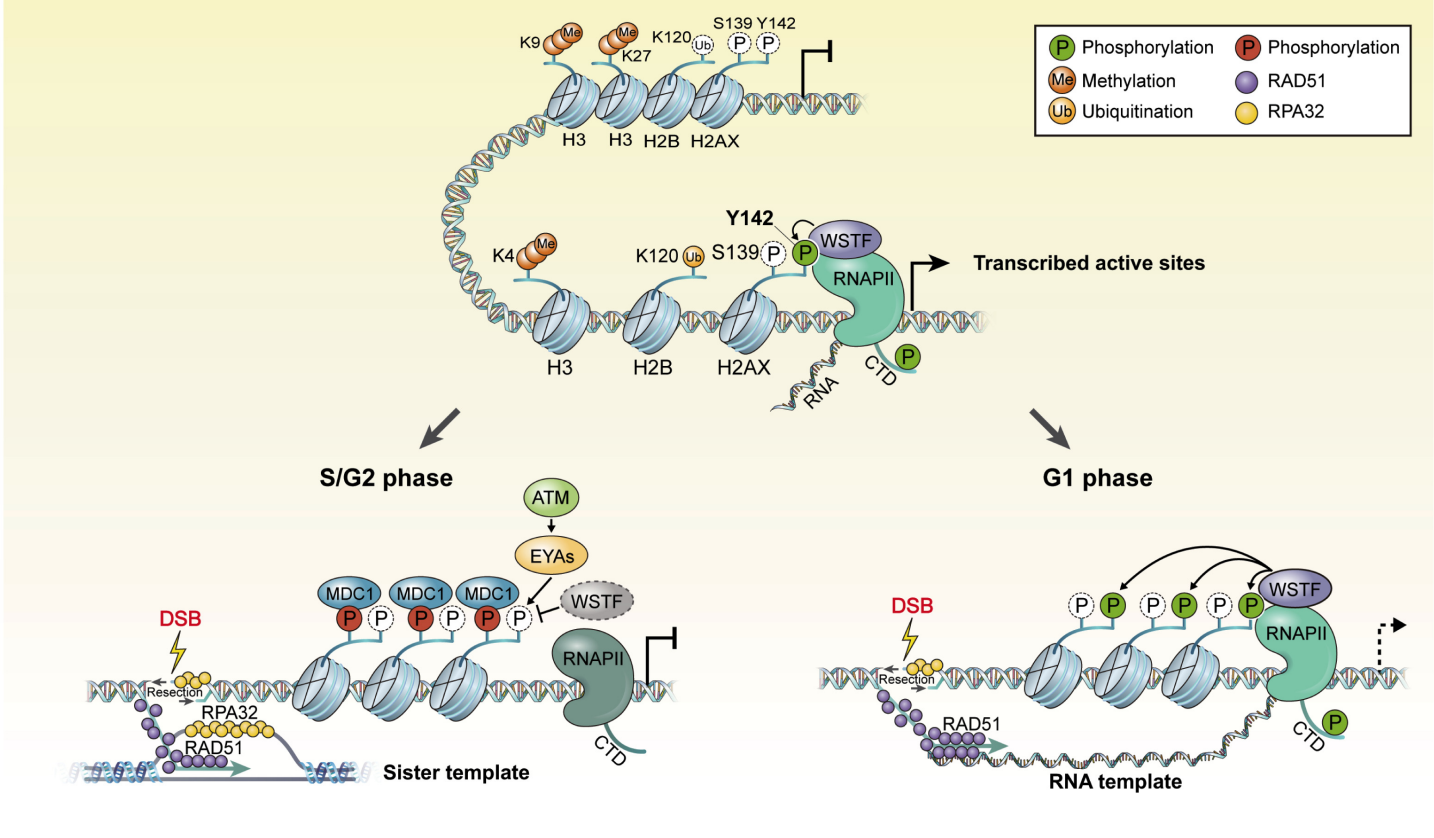
Proposed model for role of WSTF-dependent *de novo* H2AX-pY142 that regulates transcription-coupled homologous recombination repair at the G1 phase. WSTF-dependent H2AX-pY142 is coupled to RNA polymerase II (RNAPII)-mediated active transcription in proliferating cells (the upper). In the DNA damage response (DDR), removal of pre-existing H2AX-pY142 by ATM-dependent EYAs is required for γH2AX-dependent DNA damage signaling and transcriptional silencing at the DSB sites. In S/G2 phase, RPA32 and RAD51 factors are recruited at DSB sites and RAD51 utilizes sister templates as donor for homologous recombination (HR) repair (left, the lower). On the other hand, in G1 phase, the axis of *de novo* H2AX-pY142 by WSTF and RNAPII at DSB sites facilitates transcription-coupled HR (TC-HR) repair, whereby RAD51 employs RNA templates as donor for strand invasion (right, the lower).

Our observations indicate that the initial dephosphorylation of Tyr142 of H2AX by EYAs at DNA lesions is recovered at later time points, and the nascent RNA transcript (5-EU) is also coupled to *de novo* phosphorylation of H2AX on Tyr142. Finally, *de novo* Tyr142 phosphorylation-mediated active RNA transcripts by WSTF, which is linked to RNAPII activity, are required for TC-HR repair via RAD51 loading for strand invasion, using RNAs as donor templates during G1 (Figure 7). The MRN complex functions during the initial resection and trimming of proximal damage regions, and is run out from the damaged site by extensive resection. Even if interaction of the MRN complex with active RNAPII is essential to generate DDRNAs (18), it is unclear whether this phenomenon controls TC-HR repair as a late DDR event, or whether it is cell cycle-dependent. If this complex supports TC-HR repair, there is no doubt that it will be sequestered at damaged regions, but it should also dissociate from these regions via template switch with RNA transcripts through Exo1-dependent DNA extensive resection in efficient HR repair. In this case, the mechanism by which RNAPII-dependent RNA transcripts are able to be used as templates for TC-HR repair remains unknown. We hypothesize that the newly phosphorylated Tyr142 of H2AX, generated via translocation of WSTF to DSB sites, may function as a docking site of RNAPII-dependent active RNA transcripts for TC-HR repair in cell cycle-dependent manner. This hypothesis is supported by our finding that the strong interaction of WT H2AX with RNAPII dissociated under DNA damage conditions, whereas the constitutive Y142F H2AX mutant did not bind to RNAPII under normal or damaged conditions (Figure 2D). However, further studies support to elucidate the mechanism by which TC-HR repair is regulated via H2AX Tyr142 phosphorylation. Specifically, it remains to be determined if Tyr142 phosphorylation-specific active RNA transcripts or non-coding RNAs are required for TC-HR repair, and if Tyr142 phosphorylation-dependent RNAPII generates newly synthesized DDRNAs or uses non-synthesized old mRNAs or non-coding RNAs for repair. In addition, the way in which Tyr142 phosphorylation regulates TC-HR repair in active versus inactive regions should be examined.

## Supplementary Material

gkz309_Supplemental_File
