# Supplementary material for: De novo phosphorylation of H2AX by WSTF regulates transcription-coupled homologous recombination repair
Source: Nucleic Acids Res. 2019 May 2;47(12):6299–314. doi: 10.1093/nar/gkz309 (PMC6614800; doi:10.1093/nar/gkz309)
Supplement: gkz309_Supplemental_File [file gkz309_supplemental_file.pdf]

Supplementary Figure 1. Related to Figure 1.

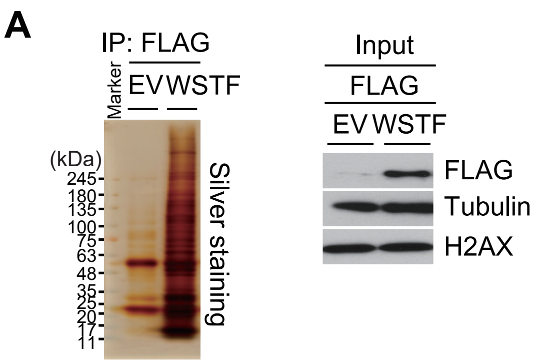

B

Protein	Fold change (log2, WSTF/EV)	Functions
**WSTF**	**27.06**	**Tyrosine-protein kinase BAZ1B**
SNF2h (SMARCA5)	3.15	SWI/SNF-related matrix-associated actin-dependent regulator of chromatin subfamily A member 5
RSF1	22.82	Remodeling and spacing factor 1
ACF1	22.78	Bromodomain adjacent to zinc finger domain 1A, BAZ1A
RPB1	24.02	RNA polymerase II subunit POLR2A
RPB3	21.78	RNA polymerase II subunit POLR2C
CDK12	25.18	Cyclin-dependent kinase 12, POLR2A phosphorylation (pS2) for transcription elongation
SPT5	23.74	Transcription elongation factor
SPT6	23.22	Transcription elongation factor

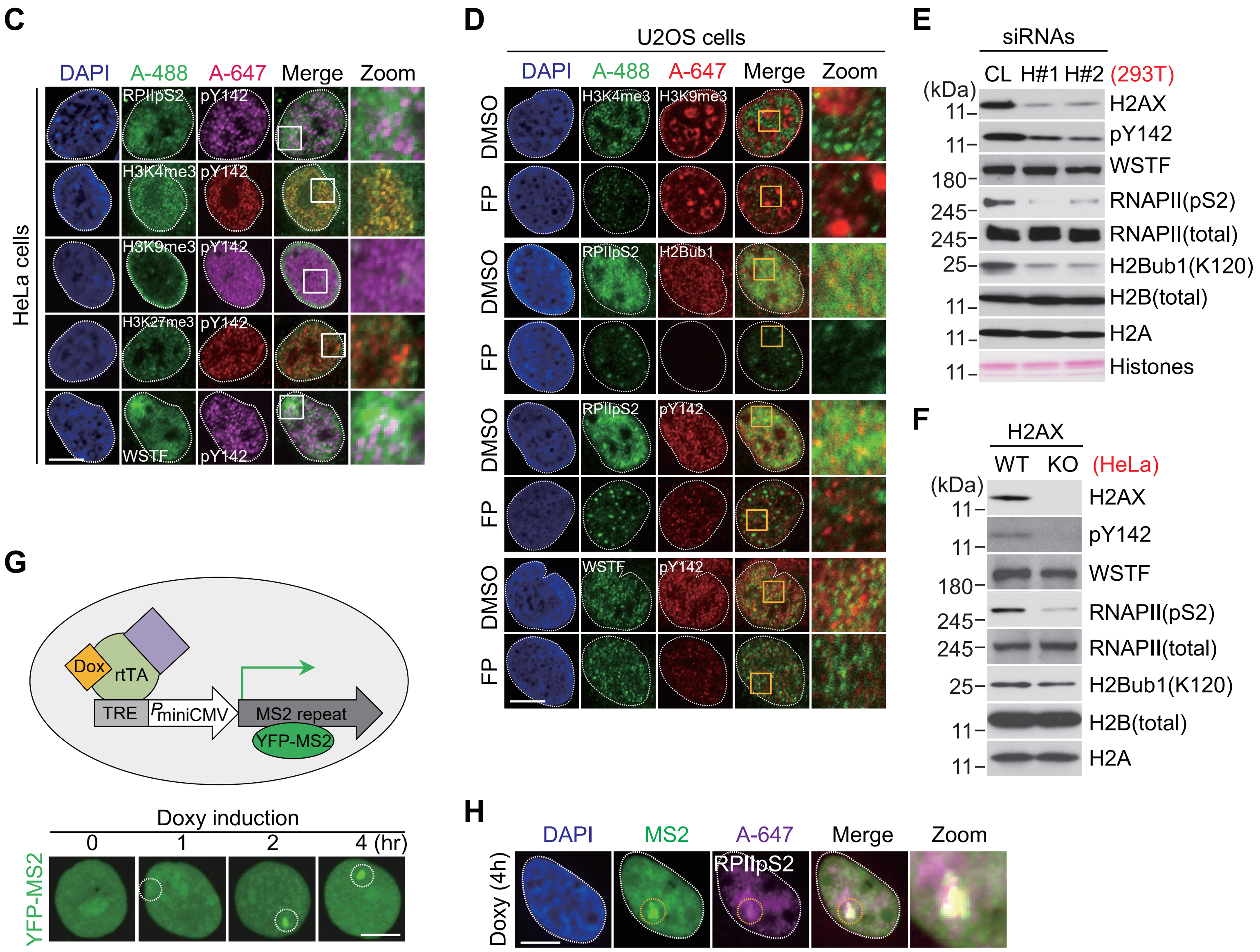

Supplementary Fig. 1. Related to Fig. 1. (**A**) To apply for mass spectrometry, 293T cells were transfected with ectopically expressed FLAG-WSTF or empty vector (EV). At 48 hr post-transfection, cell lysates were immunoprecipitated with anti-FLAG agarose. Immunoprecipitated FLAG-WSTF and its interacting proteins were confirmed by silver staining (left panel). Expression levels of FLAG-WSTF, tubulin, and H2AX were estimated by Western blots (right panels). (**B**) WSTF interacting proteins representative by mass spectrometry analysis. (**C**) Subcellular localizations of H2AX-pY142 (pY142), active markers [RNA polymerase II (RPIIpS2) and H3K4me3], inactive markers (H3K9me3 and H3K27me3) and WSTF in proliferating HeLa cells. Scale bars, 10 μm. (**D**) U2OS cells were treated with RNA polymerase II inhibitor flavopiridol (FP) or DMSO for 4 hr, and then cells were immunostained with indicated antibodies. Scale bars, 10 μm. (**E** and **F**) 293T cells were transfected with control siRNA (CL) or H2AX siRNAs (#1 or #2) for 48 hr (E), and then chromatin fractions isolated from E or H2AX wild type (WT) and knock-out (KO) HeLa cells (F) were western-blotted with indicated antibodies. (**G**) Local transcription visualizing system in single live cell. Live cells show accumulation of viral coat protein (YFP-MS2) within the reporter transcript by doxycycline (Doxy) induction in time-dependent manner. Scale bars, 10 μm. (**H**) Evaluation of active RNA polymerase II (RPIIpS2) enrichments at local transcription site after doxycycline induction (Doxy) for 4 hr. Scale bars, 10 μm.

Supplementary Figure 2. Related to Figure 3.

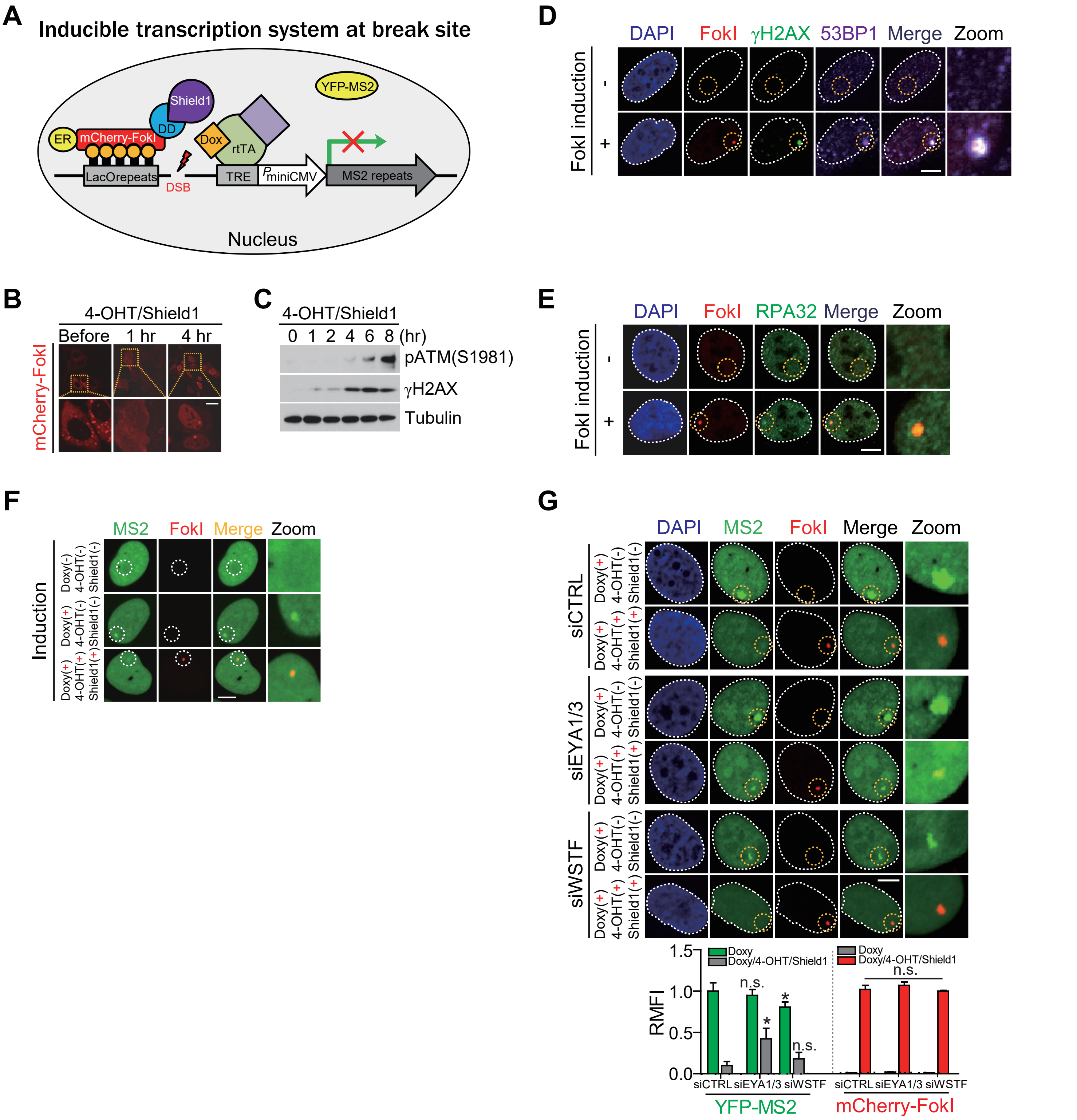

Supplementary Fig. 2. Related to Fig. 3. (**A**) A locally inducible DSB-mediated transcription system. Briefly, an accumulation of viral coat protein (YFP-MS2) within the reporter transcript near to Lac operator (LacO) is visualized by doxycycline (Doxy) induction. An stably expressed estrogen receptor (ER) fused mCherry-LacI-FokI-DD is translocated into chromosome1p3.6 locus integrated with LacO (x256 repeats) by induction of 4-OHT and Shield1 ligand, whereby mCherry-LacI-FokI makes DNA double-strand breaks (DSBs) by interacting with LacO. DD, degradation domain. (**B** and **C**) Evaluation of locally inducible DSB-mediated transcription system. The reporter cells were treated with 4-OHT and Shield1 in different time points. Expression of mCherry-LacI-FokI and its translocation to DSB sites were visualized with confocal microscope (Nikon A1) (B), and activation of DNA damage signaling factors, pATM and γH2AX, was detected by Western blots (C). Scale bars, 10 μm. (**D** and **E**) The recruitments of DNA damage repair factors 53BP1 and RPA32 were detected with anti-53BP1 (D) and anti-RPA32 (E) antibodies by FokI induction. An anti-γH2AX antibody was used for detecting DNA damage sites. Scale bars, 10 μm. (**F**) An accumulation of YFP-MS2 (MS2) and mCherry-LacI-FokI (FokI) at DSB sites by induction of doxycycline (Doxy) and/or 4-OHT and Shield1. Scale bars, 10 μm. (**G**) The reporter cells were transfected with control, EYA1/EYA3, or WSTF siRNAs for 48 hr. An accumulation of YFP-MS2 (MS2) at DSB sites was induced by treatment with doxycycline (Doxy) for 4 hr. At the same time, DNA double strand breaks were occurred by FokI induction. Scale bars, 10 μm. Quantitative analysis for the fluorescence intensity of YFP-MS2 and FokI at DSB sites. The plotted values represent mean ± SEM of more than 50 individual cells. n.s. means not significant.

Supplementary Figure 3. Related to Figure 4.

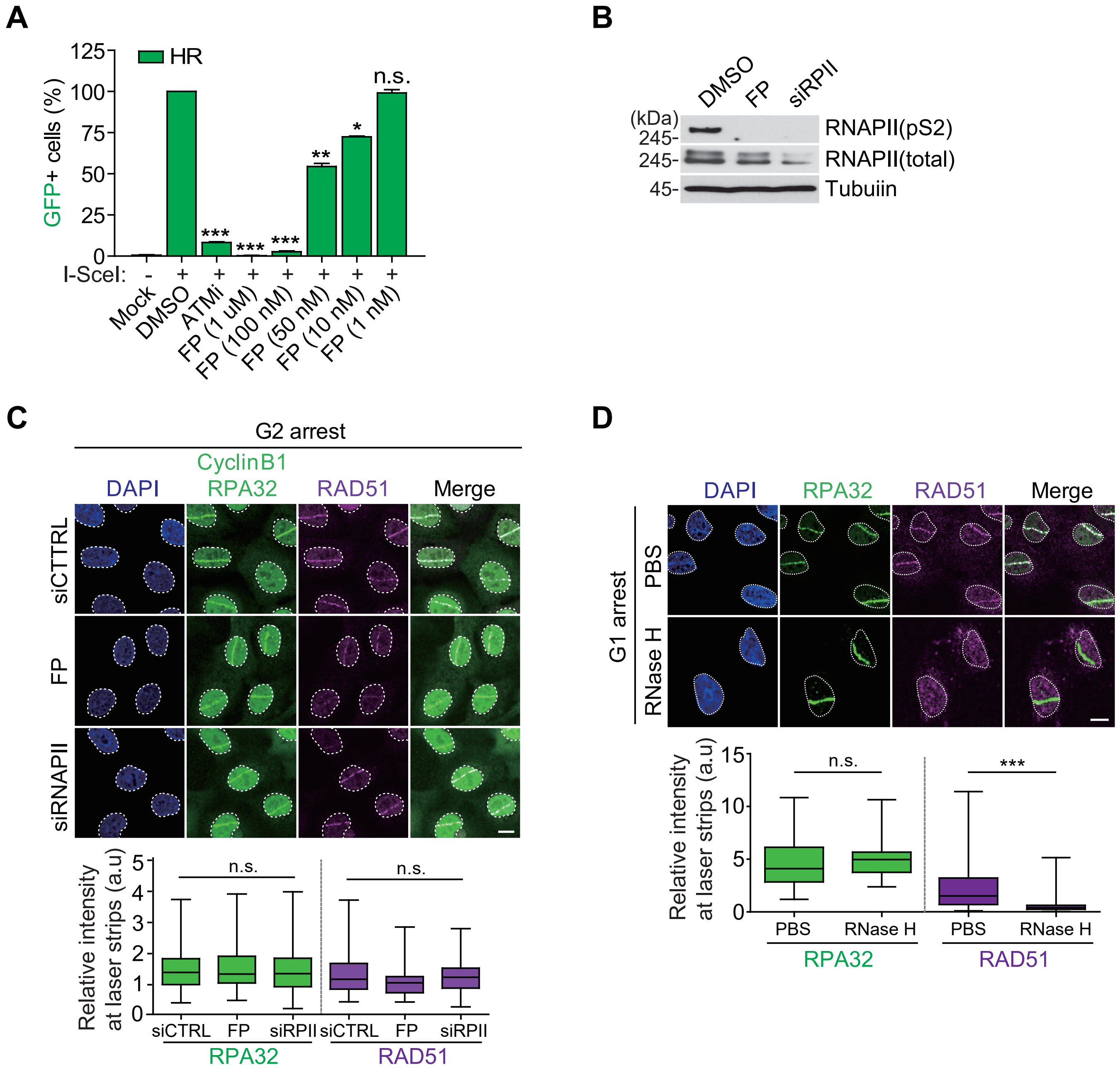

Supplementary Fig. 3. Related to Fig. 4. (**A**) Comparative analysis of HR repair into HR reporter cells. ATM inhibitor (ATMi) and different doses of flavopiridol (FP) were treated after transfection with I-SceI plasmid. Data represent mean ± SEM of three independent experiments. n.s. means not significant. (**B**) Expression levels of RNAPII and RNAPIIpS2 by depletion or inhibition of RNAPII. (**C**) HeLa cells were transfected with control or RNAPII-targeting siRNA and released to arrest at G2 phase by double thymidine block. Flavopiridol (FP) was treated before 1 hr microirradiation. At 2 hr post-microirradiation, fixed cells were immunostained with indicated antibodies. An anti-Cyclin B1 antibody was used for G2-specific. Scale bars, 10 μm. Data represent mean ± SEM of more than 20 cells. n.s. means not significant. (**D**) G1-arrested HeLa cells were treated with RNase H for 10 min at 2 hr post-microirradiation. Then, fixed cells were immunostained with indicated antibodies. Scale bars, 10 μm. Data represent mean ± SEM of three independent experiments. n.s. means not significant.

Supplementary Figure 4. Related to Figure 5.

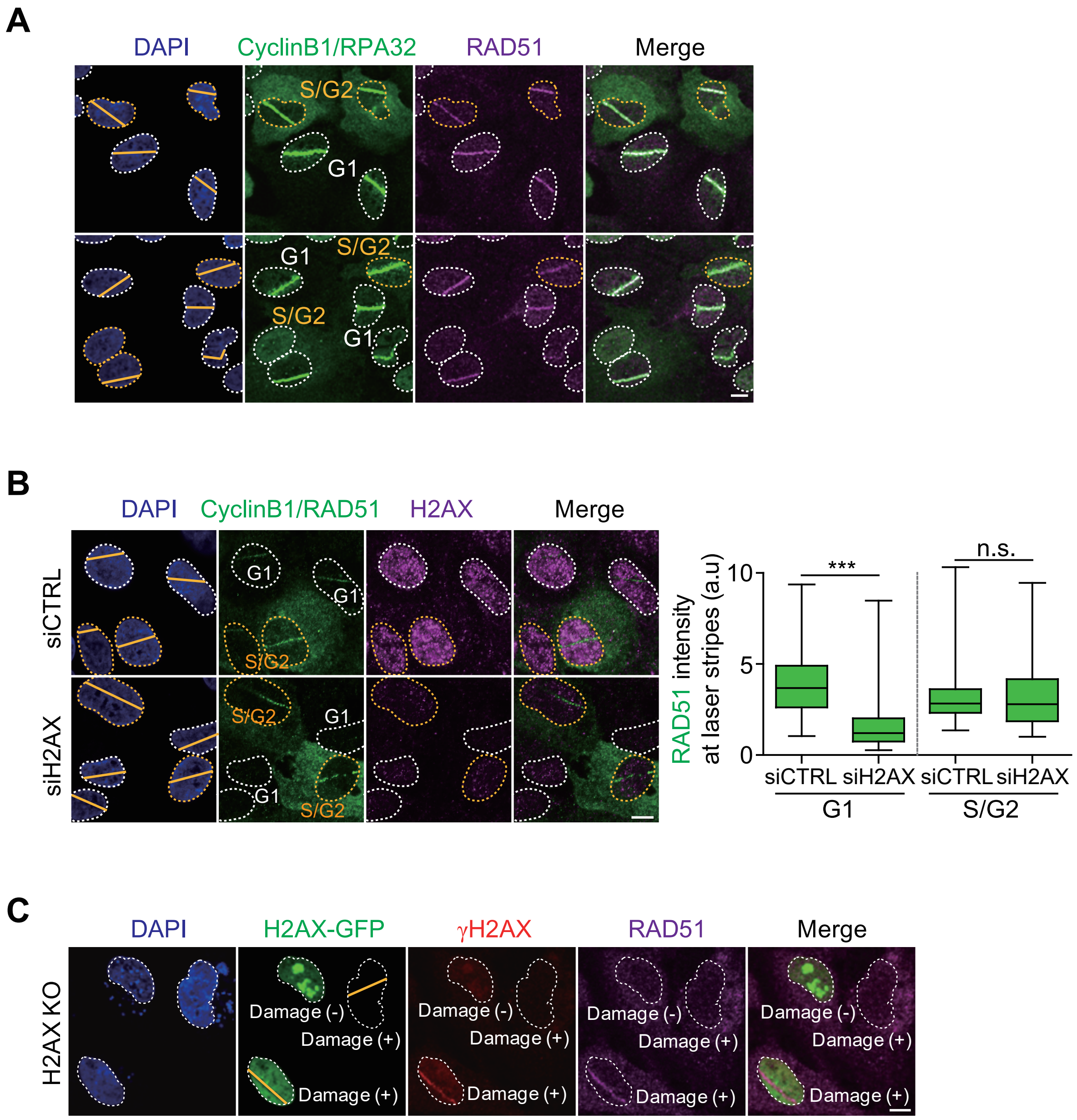

Supplementary Fig. 4. Related to Fig. 5. (**A**) U2OS cells were applied to microirradiation. Fixed cells were immunostained with anti-RPA32 and anti-RAD51 antibodies at 2 hr post-microirradiation. An anti-Cyclin B1 antibody was used for detecting S/G2 phasic cells. Scale bars, 10 μm. (**B**) U2OS cells were transfected with control or H2AX-targeting siRNA. After 2 hr, microirradiated cells were fixed and immunostained with anti-RAD51 and anti-H2AX antibodies. An anti-Cyclin B1 antibody was used for S/G2-specific. Scale bars, 10 μm. Data represent mean ± SEM of three independent experiments. n.s. means not significant. (**C**) HeLa H2AX KO cells were transfected with H2AX-GFP wild type. At 48 hr post-transfection, cells were applied to microirradiation. Fixed cells were immunostained with anti-γH2AX and anti-RAD51 antibodies at 2 hr post-microirradiation. Scale bars, 10 μm.

Supplementary Figure 5. Related to Figure 6.

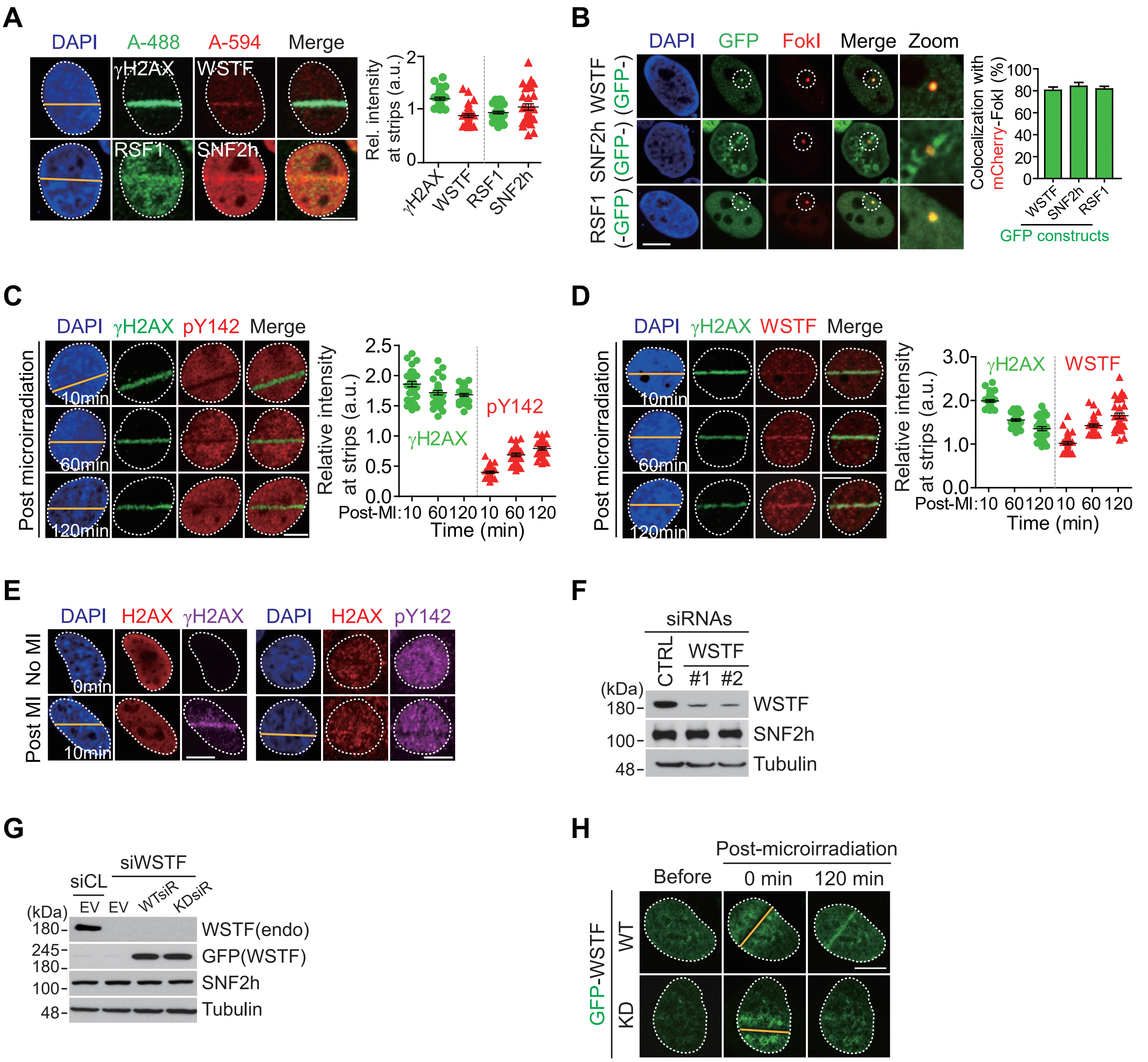

Supplementary Fig. 5. Related to Fig. 6. (**A**) U2OS cells were fixed and immuno-stained with anti-γH2AX and anti-WSTF or anti-RSF1 and anti-SNF2h antibodies at 10 min post-microirradiation. Scale bars, 10 μm. Data represent mean ± SEM of three independent experiments. (**B**) U2OS 2-6-3 cells were transfected with GFP-fused WSTF, SNF2h, and RSF1 in combination with mCherry-LacI-FokI plasmid for 48 hr. Cells were fixed and stained with 4',6-diamidino-2-phenylindole (DAPI) solution. The colocalization of GFP-fused proteins and FokI was detected by confocal microscope (Nikon A1). Scale bars, 10 μm. Data represent mean ± SEM of more than 50 cells. (**C**-**E**) U2OS cells were microirradiated and fixed at indicated time points. Fixed cells were immunostained with anti-γH2AX and anti-pY142 (C), anti-γH2AX and anti-WSTF (D), or anti-H2AX and anti-γH2AX or anti-pY142 (E) antibodies. Scale bars, 10 μm. Data represent mean ± SEM of three independent experiments. (**F**) Expression levels of endogenous WSTF by WSTF-targeting siRNAs (#1 and #2). (**G**) Expression levels of endogenous WSTF and ectopic WSTF resistant WSTF-targeting siRNA into U2OS cells. (**H**) The live cell images of accumulation of GFP-fused WSTF wild type (WT) and kinase-dead mutant (KD) at laser stripes in different time points (0 min and 120 min) in U2OS cells.

Supplementary Table 1. List of antibodies used in this study.

Antibody	Company	Clone, catalog no.
WSTF	Fitzgerald	10R-1247
WSTF	Santa Cruz Biotechnology	BAZ1H4H9, sc-81426
WSTF	Sigma-Aldrich	W3766
γH2AX	Merk	JBW301, 05-636
γH2AX	GeneTex	GTX127340
H3K4me3	Abcam	ab1012
H3K9me3	Abcam	ab8898
H3K9me3	Active Motif	2AG-6F12-H4, 39286
pY142	Abcam	ab94602
pY142	Merk	07-1590
pY142	Thermo Fisher Scientific	PA5-40153
RNA polymerase II (total)	BioLegend	8WG16
RNA polymerase II (pS2)	BioLegend	H5
H2Bub1 (K120)	Cell Signaling Technology	D11, 5546
H2B (total)	Cell Signaling Technology	D2H6, 12364
H2AX	Cell Signaling Technology	D17A3, 7631
H2AX	LSBio	3F4, LS-C197392
Tubulin	Santa Cruz Biotechnology	B-5-1-2, sc-23948
H3	Abcam	ab1791
FLAG	Sigma-Aldrich	M2, F3165
H2A	Abcam	ab18255
H3K27me3	Abcam	ab6002
EYA1	Santa Cruz Biotechnology	L-19, sc-15094
EYA3	Abcam	ab22835
GFP	Santa Cruz Biotechnology	B-2, sc-9996
MDC1	Abcam	ab11169
RPA32	Abcam	9H8, ab2175
RAD51	Merk	Ab-1, PC130
RAD51	GeneTex	N1C2, GTX100469
RAD51	Novus	14B4, NB100-148
53BP1	Cell Signaling Technology	4937
SNF2h	Merk	8F9.1, MABE120
pATM(S1981)	Abcam	ab36810
Cyclin E	Santa Cruz Biotechnology	M-20, sc-481
Cyclin B1	Santa Cruz Biotechnology	GNS1, sc-245
CtIP	Santa Cruz Biotechnology	D-4, sc-271339

Supplementary Table 2. List of siRNAs used in this study.

Gene	siRNA Sequences	Targeting region	Supplier (http://www.genolution1.com)
WSTF (#1)	5'-GAACAGGAAGUUGCUGAGC-3’	CDS	Genolution
WSTF (#2)	5'-GAUAGUUCGAUACUUUAUAUU-3’	CDS	Genolution
H2AX (#1)	5’-CACCCAGGCCUCCCAGGAGUACUAA-3’	CDS	Genolution
H2AX (#2)	5’-GGGACGAAGCACUUGGUAA-3’	3' UTR	Genolution
H2AX (#3)	5’-GGAAAGAGCUGAGCCGCUUUU-3’	3' UTR	Genolution
EYA1	5’-CAGGAAAUAAUUCACUCACAA-3’	CDS	Genolution
EYA3 (#1)	5’-GGAUUAUCCCACCUAUACUUU-3’	CDS	Genolution
EYA3 (#2)	5’-GCUCGAUUGGUUCUACUGU-3’	3' UTR	Genolution
RNAPII (#1)	5’-CGUACAAUGCAGACUUUGAUU-3’	CDS	Genolution
RNAPII (#2)	5’-GAAGUUUAAAUAAAGUUUAUU-3’	3' UTR	Genolution

Supplementary Table 3. List of plasmids used in this study.

Plasmid	Supplier	Catalog No.
pcDNA-DEST-FLAG_H2AX_WT	in this study	N/A
pcDNA-DEST-FLAG_H2AX_YF	in this study	N/A
pcDNA-DEST-FLAG_H2AX_YE	in this study	N/A
pcDNA-DEST47_H2AX_WT	in this study	N/A
pcDNA-DEST47_H2AX_YF	in this study	N/A
pcDNA-DEST47_H2AX_YE	in this study	N/A
pcDNA-DEST47_H2AX_SA	in this study	N/A
pcDNA-DEST47_H2AX_SE	in this study	N/A
pcDNA-DEST53_WSTF_WT	in this study	N/A
pcDNA-DEST53_WSTF_WT_siR	in this study	N/A
pcDNA-DEST53_WSTF_KD	in this study	N/A
pcDNA-DEST53_WSTF_KD_siR	in this study	N/A
pcDNA-DEST53_EYA1	in this study	N/A
pcDNA-DEST53_EYA3	in this study	N/A
pcDNA-DEST53 (EV)	Thermo Fisher Scientific	#12288015
pcDNA-DEST47 (EV)	Thermo Fisher Scientific	#12281010
pcDNA-DEST-FLAG (EV)	in this study	N/A
I-SceI plasmid	gift from Jeremy Stark	N/A
pcDNA-DEST-FLAG_WSTF	in this study	N/A
pcDNA-DEST-FLAG_EYA3	in this study	N/A
pEGFP-N1_RSF1	in this study	N/A
pEGFP-C1_SNF2h	in this study	N/A
pcDNA-DEST53_RNF2	in this study	N/A
FLAG-RNA Polymerase II_WT	Addgene	#35175
FLAG-RNA Polymerase II _ΔCTD	Addgene	#35176

Supplementary Table 4. List of mutagenesis primer sets used in this study.

Plasmid (pCR8/GW-TOPO)	Forward primers for mutagenesis	Reverse primers for mutagenesis	Supplier (http://www.macrogen.com)
WSTF-KD (C338A)	5'-CCACTAAATCCTAAGTTATGGGCTCACGTACACTTGAAGAAGTC-3'	5'-GACTTCTTCAAGTGTACGTGAGCCCATAACTTAGGATTTAGTGG-3'	MACROGEN
WSTF_WT_ siRNA-resistant	5'-GAACAAGAGGTAGCTGAGCTTTTGAA-3'	5'-TTCAAAAGCTCAGCTACCTCTTGTTC-3'	MACROGEN
WSTF_KD_ siRNA-resistant	5'-GAACAAGAGGTAGCTGAGCTTTTGAA-3'	5'-TTCAAAAGCTCAGCTACCTCTTGTTC-3'	MACROGEN
H2AX_WT sequence	*5'-AAGGCCACCCAGGCCTCCCAGGAGTAC-3'*	*5'-GTACTCCTGGGAGGCCTGGGTGGCCTT-3'*	*Reference sequences (S139/Y142)*
H2AX_S139A	5'-AAGGCCACCCAGGCCGCCCAGGAGTAC-3'	5'-GTACTCCTGGGCGGCCTGGGTGGCCTT-3'	MACROGEN
H2AX_S139E	5'-AAGGCCACCCAGGCCGAACAGGAGTAC-3'	5'-GTACTCCTGTTCGGCCTGGGTGGCCTT-3'	MACROGEN
H2AX_Y142F	5'-AAGGCCACCCAGGCCTCCCAGGAGTTC-3'	5'-GAACTCCTGGGAGGCCTGGGTGGCCTT-3'	MACROGEN
H2AX_Y142E	5'-AAGGCCACCCAGGCCTCCCAGGAGGAA-3'	5'-TTCCTCCTGGGAGGCCTGGGTGGCCTT-3'	MACROGEN
